# Complete chloroplast genome sequence of *Pachystachys lutea* Nees: genome structure, adaptive evolution, and phylogenetic relationships

**DOI:** 10.1186/s12863-025-01380-9

**Published:** 2025-11-19

**Authors:** Changmei Du, Yan Dong, Haishuo Gao, Tingting Fang, Jianhua Yue, Yan Zhang

**Affiliations:** 1 School of Horticulture, Xinyang Agriculture and Forestry University, Xinyang, 464100 China; 2 School of Forestry, Xinyang Agriculture and Forestry University, Xinyang, 464100 China; 3 Xinyang Camellia Oleifera Industry Development Center, Xinyang, 464100 China; 4 Department of Plant Science and Technology, Shanghai Vocational College of Agriculture and Forestry, Shanghai, 201699 China

**Keywords:** *Pachystachys lutea*, Acanthaceae, Chloroplast genome, Comparative analysis, Phylogenetic analysis

## Abstract

**Background:**

*Pachystachys lutea* Nees is a typical species of the family Acanthaceae, native to tropical South America. As an evergreen shrub, it has found extensive application in landscape greening due to its unique ornamental value. However, there are currently no phylogenetic and genetic studies on the chloroplast (cp.) genome of *P. lutea*.

**Results:**

This study characterized the cp. genome of *P. lutea* using high-throughput sequencing technology and analyzed its structural features and phylogenetic position using bioinformatics methods. The results indicated that the cp. genome had a high degree of conservation in gene structure and gene content, with a typical quadripartite structure. Its total length is 151,574 bp and the total GC content is 38.18%. A total of 132 genes were annotated, including 87 protein-coding genes (PCGs), 37 tRNAs and eight rRNA genes. Through the comparative analysis, the diversity and variation of large single-copy (LSC) and small single-copy (SSC) regions were significantly higher than those of inverted repeat (IR) regions. Genes with high nucleotide polymorphism, such as *rps19*, *ycf1*, and *ndhF* provided potential reference loci for molecular identification within the *P. lutea*. The phylogenetic analysis showed that the *P. lutea* and *Clinacanthus nutans* forms a sister group with 100% bootstrap value, which proves that *P. lutea* develops conservatively in the course of evolution.

**Conclusion:**

This paper for the first time reports the phylogenetic study of the complete cp. genome within the genus *Pachystachys*. The study provides a theoretical basis for the research on genetic diversity, molecular markers, and species identification of plants in the Acanthaceae family. It enriches the genetic information and supports the evolutionary relationships among plants in this family.

**Supplementary Information:**

The online version contains supplementary material available at 10.1186/s12863-025-01380-9.

## Introduction

**Fig. 1:**
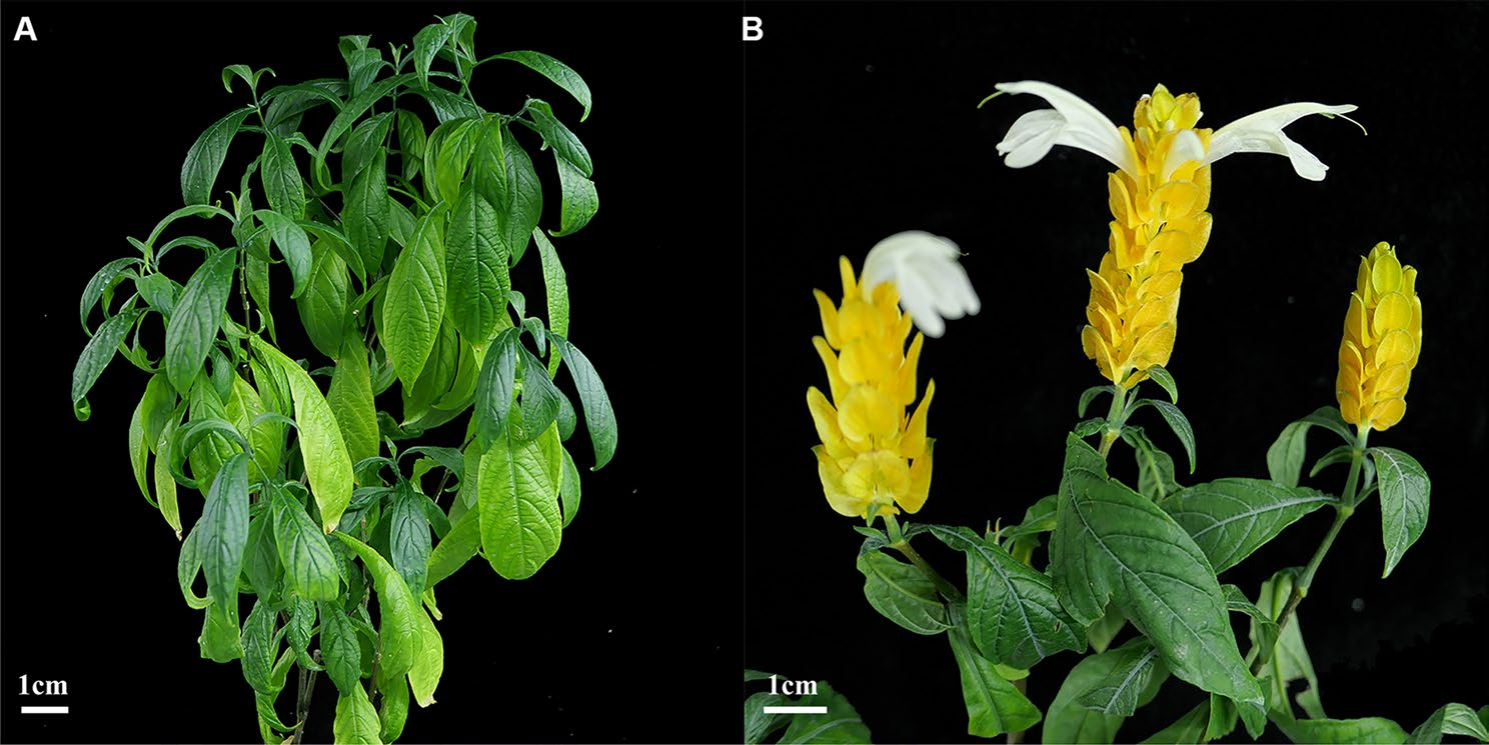
Plant morphological characteristics (**A**) and the floral organ morphology (**B**) of *P. lutea*

The Acanthaceae family contains approximately 250 genera and more than 4000 species [1, 2], mainly distributed in tropical and subtropical areas [3, 4]. Approximately 50 genera and more than 400 species are distributed in China [5]. Many species of the Acanthaceae family are remarkable flowering ornamental plants, characterized by diverse forms, vivid colors, and extended ornamental periods. *Pachystachys lutea* Nees is one of them, is a perennial evergreen flowering shrub that prefers high temperatures, high humidity and sunny environments and is mainly distributed in the southern areas of the Yangtze River in China. The flower has a unique shape and blooms continuously throughout the year in warm environments (the greenhouse of Shanghai Chenshan Botanical Garden), so it is often planted as an ornamental plant. *P. lutea* has very large spikelike inflorescences at the top of each new branch, and the golden bracts are stacked and spread out with small white flowers like shrimp bodies (Fig. 1). Furthermore, its roots, leaves, and flowers are also used for medicinal purposes, treating pneumonia [6], diarrhea, worms [7], etc., and are the important medicinal plant resources to be developed. In addition, it has economic value. The leaves serve as the source of endophytic fungi [8, 9] and blue dye [10], and nectar has the potential to serve as a substitute for sugar production in the future [11]. Currently, there are relatively few species within the genus *Pachystachys* that have been named, yet its outstanding ornamental characteristics have made *P. lutea* highly recommended in China, where it has become an extremely valuable ornamental plant resource, widely loved by the public, and effectively enhancing the diversity of landscape species applications.

The chloroplast (cp.), an important organelle in green plants [12], houses the cp. genome which is of great significance in plant evolutionary biology research. It presents remarkable benefits due to its genetic stability, well-preserved genome structure, and faster rate of evolutionary change compared to mitochondria. Consequently, the cp. genome is widely utilized in the study of genetic relationships and species evolution between different species [13, 14]. Despite its significant ornamental and medicinal value, there is no high-quality reference cp. genome that has been published or made publicly available, which limits our understanding of its evolutionary history and genetic traits.

*P. lutea* is a species within the genus *Pachystachys* of the family Acanthaceae. Research on *P. lutea* has mainly focused on its horticultural applications, cultivation techniques, and medicinal properties, leaving its genetic and phylogenetic aspects largely unexplored. Nevertheless, scant knowledge exists regarding its phylogenetic relationship and evolutionary history, primarily due to the dearth of research on the modern taxonomic system, especially in the scarcely explored realm of molecular evolution. Scotland and Vollesen (2000) [15], initially applied molecular systematics in conjunction with floral organ development to establish a classification system for the family Acanthaceae. Through a comprehensive study of *trnL-trnF* and internal transcribed spacers (ITS), it was demonstrated that *Pachystachys* shares a common lineage with *Henrya*, *Carlowrightia*, *Anisacanthus*, and *Tetramurium*, and this branch is further grouped with the one encompassing *Dicliptera* and *Justicia*. However, the position of *P. lutea* within the Acanthaceae requires further validation.

This study aims to: (1) analyze the structural characteristics of the cp. genome of *P. lutea*, (2) investigate its adaptive evolutionary traits, and (3) clarify its phylogenetic relationships with closely related species.

To achieve these aims, we employed molecular biology and high-throughput sequencing techniques to characterize the cp. genome of *P. lutea*. This involved using the Illumina NovaSeq 6000 platform for sequencing and a suite of bioinformatics tools for genome assembly, annotation, and analysis. We also compared the results with those obtained from traditional morphological taxonomic methods, aiming to provide a more comprehensive perspective for its taxonomic study. Studies at molecular level can provide more precise genetic information. By filling this research gap, we aim to lay a solid foundation for more accurately determining its taxonomic position and facilitating future research endeavors.

## Results

### Genome structure of the *P. lutea* cp. genome

**Fig. 2:**
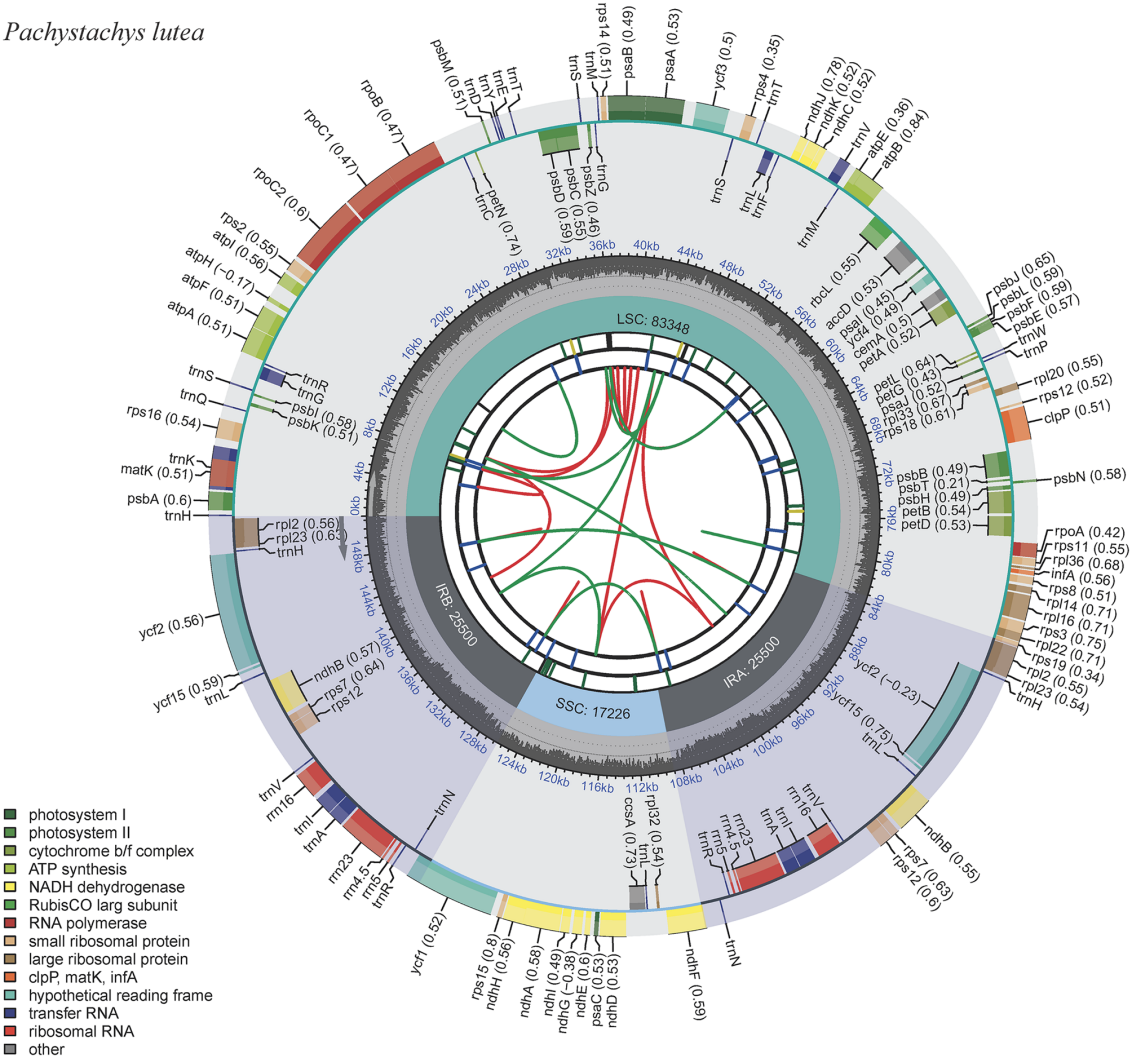
The chloroplast genome map of *P. lutea*. Genes outside the circle are transcribed clockwise, while genes inside the circle are transcribed counterclockwise. Different functional groups are represented by different colors. The darker and lighter gray in the inner indicated the GC and AT content, respectively

In this study, we assembled and annotated the complete cp. genome, analyzed the sequencing depth, which reached an average of 3,012× sequencing depth (Fig. S1). Compared with that of other species [16, 17], this higher sequencing depth has enhanced the reliability of gene annotation and structural analysis. The accuracy of the assembly and annotation of *P. lutea* cp. genome was evaluated, respectively (Fig. S2; Fig. S3). The complete cp. genome of *P. lutea* was 151,574 bp in length (Fig. 2), and it had a typical quadripartite structure with junction regions: a large single-copy (LSC) region of 83,348 bp, a small single-copy (SSC) region of 17,226 bp, and a pair of inverted repeat (IR) regions (IRa and IRb) of 25,500 bp each. The overall GC content of the complete *P. lutea* cp. genome was 38.18%, and the corresponding values in the LSC, SSC, and IR regions were 36.21%, 32.36%, and 43.36%, respectively. The complete cp. genome was found to contain 132 genes, which comprised 87 protein-coding genes (PCGs), 37 tRNA genes, and eight rRNA genes (Table 1). Nine PCGs, six tRNA genes, and four rRNA genes were duplicated in the IR regions. Nineteen genes contained two exons, and four genes (*clpP*, *ycf3*, and two *rps12*) contained three exons. Among 87 PCGs, 45 photosynthesis genes, 29 genes related to self-replication, six other genes and seven genes with unknown function were identified.

**Table 1:** Gene function statistics table of chloroplast genome in *P. lutea*

Gene category	Gene function	Gene name
Photosynthesis gene	Subunits of photosystem I	*psaA*,* psaB*,* psaC*,* psaI*,* psaJ*
	Subunits of photosystem II	*psbL*,* psbZ*,* psbM*,* psbN*,* psbA*,* psbB*,* psbC*,* psbD*,* psbE*,* psbF*,* psbT*,* psbH*,* psbI*,* psbJ*,* psbK*
	Subunits of NADH-dehydrogenase	*ndhG*,* ndhH*,* ndhI*,* ndhJ*,* ndhK*, ndhA*, ndhB*(2),* ndhC*,* ndhD*,* ndhE*,* ndhF*
	Subunits of cytochrome b/f complex	*petL*,* petN*,* petA*, petB*, petD*,* petG*
	Subunit for ATP synthase	*atpI*,* atpA*,* atpB*,* atpE*, atpF*,* atpH*
	Large subunit of rubisco	*rbcL*
Self-replication	Large subunit of ribosome	*rpl20*,* rpl22*,* rpl32*,* rpl23(2)*,* rpl14*,* rpl33*, rpl16*,* rpl36*, rpl2*(2)
	Small subunit of ribosome	*rps11*,* rps14*,* rps15*, rps16*,* rps2*,* rps3*,* rps18*,* rps4*,* rps19*,* rps7(2)*,* rps8*, rps12**(2)
	DNA dependent RNA polymerase	*rpoA*,* rpoB*, rpoC1*,* rpoC2*
	rRNA gene	*rrn5(2)*,* rrn4.5(2)*,* rrn16(2)*,* rrn23(2)*
	tRNA gene	*trnR-UCU*,* trnE-UUC*,* trnT-GGU*,* trnS-GGA*,* trnV-GAC(2)*,* trnR-ACG(2)*, trnL-UAA*,* trnG-GCC*,* trnD-GUC*,* trnY-GUA*,* trnP-UGG*,* trnM-CAU*,* trnL-CAA(2)*,* trnS-GCU*,* trnW-CCA*,* trnF-GAA*,* trnT-UGU*,* trnS-UGA*, trnV-UAC*, trnG-UCC*,* trnL-UAG*, trnI-GAU*(2),* trnH-GUG*,* trnH-CAU(2)*,* trnQ-UUG*,* trnN-GUU(2)*, trnK-UUU*, trnA-UGC*(2),* trnC-GCA*
Other genes	Translational initiation factor	*infA*
	Maturase	*matK*
	Protease	clpP**
	Envelope membrane protein	*cemA*
	Subunit of Acetyl-carboxylase	*accD*
	C-type cytochrome synthesis gene	*ccsA*
Unknown gene	Open reading frames (ORF, ycf)	*ycf1*,* ycf2(2)*, ycf3**,* ycf4*,* ycf15(2)*

Notes: Gene *: Gene with one intron and two exons; Gene **: Gene with two introns and three exons; Gene (2): Number of copies of multi-copy genes

### Simple sequence repeats analysis

**Fig. 3:**
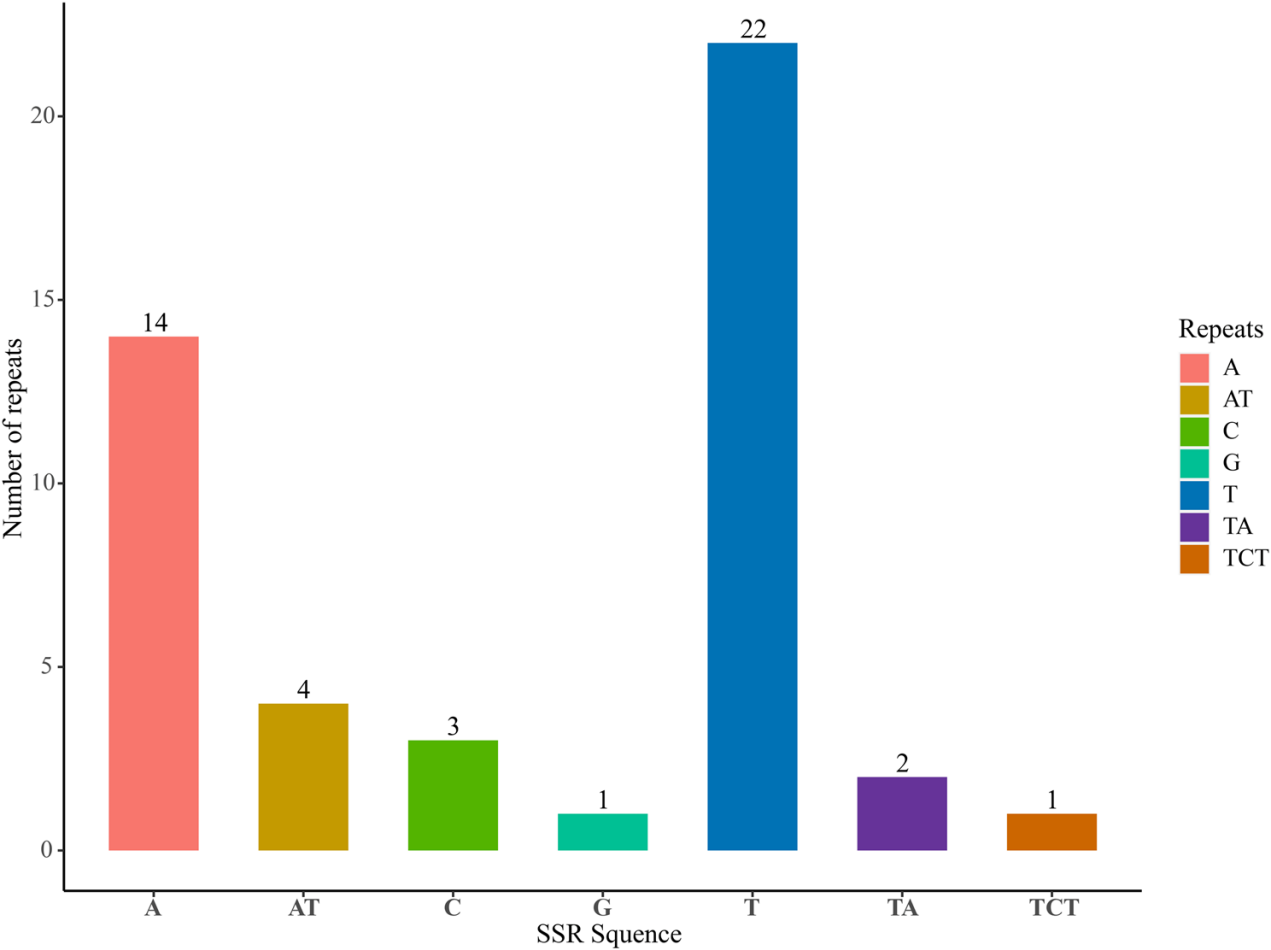
SSRs analysis of chloroplast genome in *P. lutea*. The x-axis represents the types of repetitive sequences in base pairs, while the y-axis indicates the number of SSRs found within each category

The simple sequence repeats (SSRs) analysis identified 47 SSRs in the *P. lutea* cp. genome, which mainly belonged to three types: mononucleotides, dinucleotides, and trinucleotides. Tetranucleotide, pentanucleotide, and hexanucleotide types were not found. Among them, the mononucleotides repeat sequence (A/C/T/G) type was the most abundant, with 40, which were mainly dominated by A and T bases; there were six dinucleotide repeat sequences (AT/TA), and one trinucleotide repeat sequence (TCT) (Fig. 3). Out of 47 SSR loci, 37 were concentrated in the LSC region, eight were located in the SSC region, and only two were located in the IR regions (Table S1).

### Codon preference analysis

**Fig. 4:**
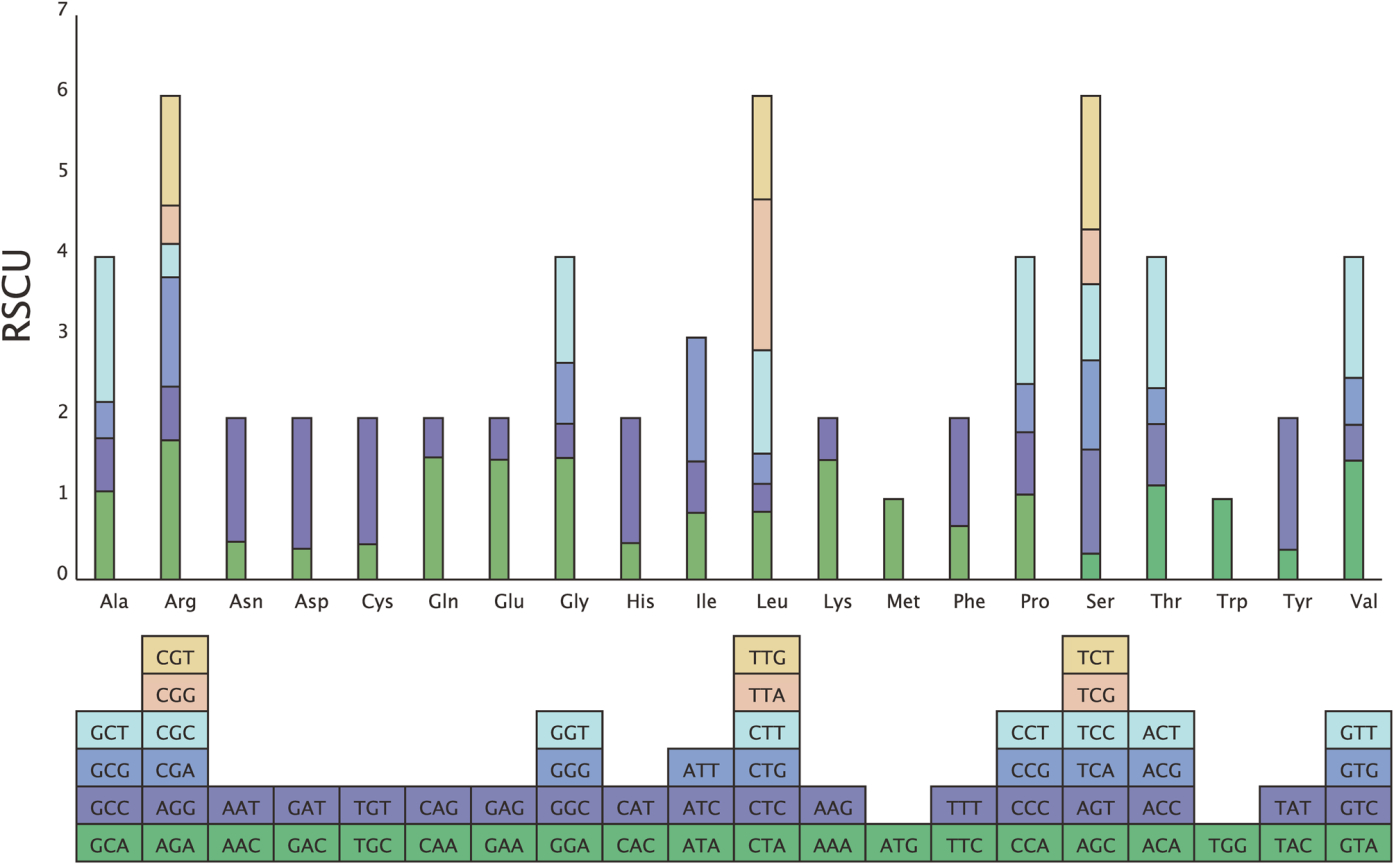
Codon usage mode of chloroplast genes in *P. lutea*. Boxes below the graphs represent all codons encoding each amino acid, with the colors of the histograms corresponding to the colors of the codons

**Fig. 5:**
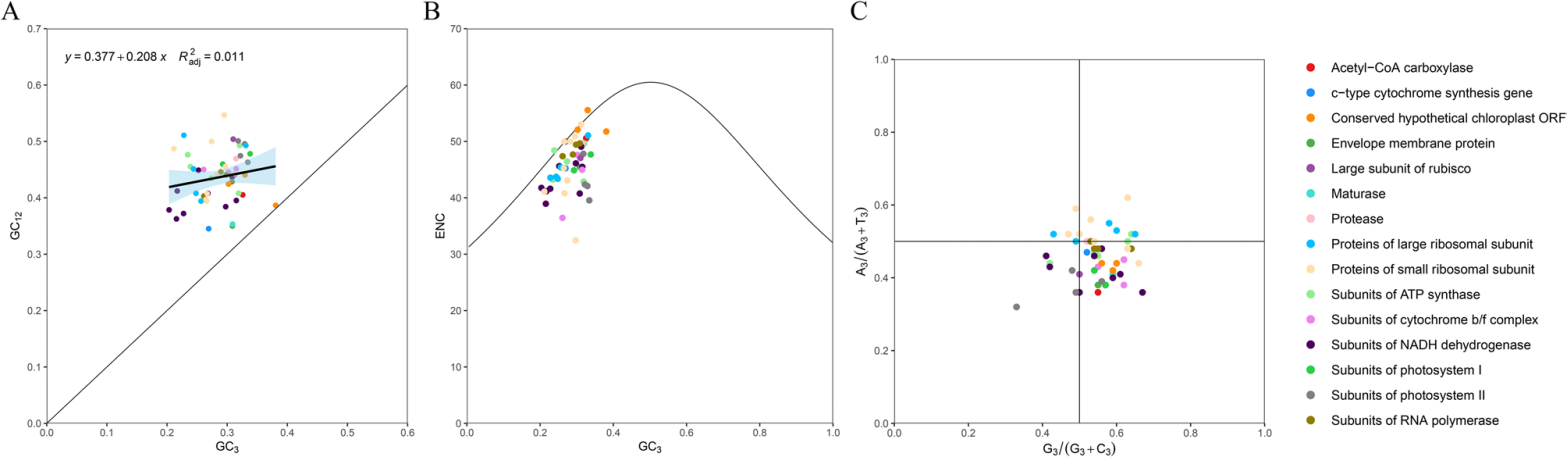
The analysis of evolutionary forces of chloroplast genes in *P. lutea*. (**A**) Neutral-plot analysis (GC3 vs. GC12). (**B**) ENC-plot analysis (GC3 vs. ENC). (**C**) PR2-plot analysis (G3/ (G3 + C3) vs. A3/ (A3 + T3)). G3/ (G3 + C3): the probability of G when the third base of codon is G or C. A3/ (A3 + T3): the probability of A when the third base of the codon is A or T

A total of 24,537 codons in 87 PCGs of the cp. genome of *P. lutea* were participated in translation protein expression. Codons with a relative synonymous codon usage (RSCU) over 1 are thought to be favored by amino acids. Overall, 31 codons had an RSCU >1.0, with 29 codons ending in A or T and two codons ending in G or C. In addition, among the amino acid codes, leucine (Leu) had the highest encoding rate, with six synonymous codon codes (TTA, TTG, CTT, CTC, CTA, CTG), with a total of 2,629. The encoding rate of cysteine (Cys) was low, and there were two synonymous codons (TGT, TGC), totaling 279. methionine (Met) and tryptophan (Trp) had only one codon, while the rest of the amino acids had two to six codons (Fig. 4; Table S2). In addition, the effective number of codons (ENC) of the PCGs was 45.83, and the GC1, GC2 and GC3 contents were 47.20%, 40.07% and 28.62%, respectively. Neutral-plot analysis showed that all genes were located above the diagonal line, indicating a weak correlation between GC12 (average of GC1 and GC2) and GC3; ENC-plot analysis showed that most of the genes were located below the standard curve and far away from the curve; and PR2-plot analysis showed that the genes were unevenly distributed in the four regions, and most of the genes were located in the lower right of the plot (Fig. 5). These results indicated that the codon preference of *P. lutea* was influenced more by natural selection [18–20].

### IR/SC boundary comparison analysis

**Fig. 6:**
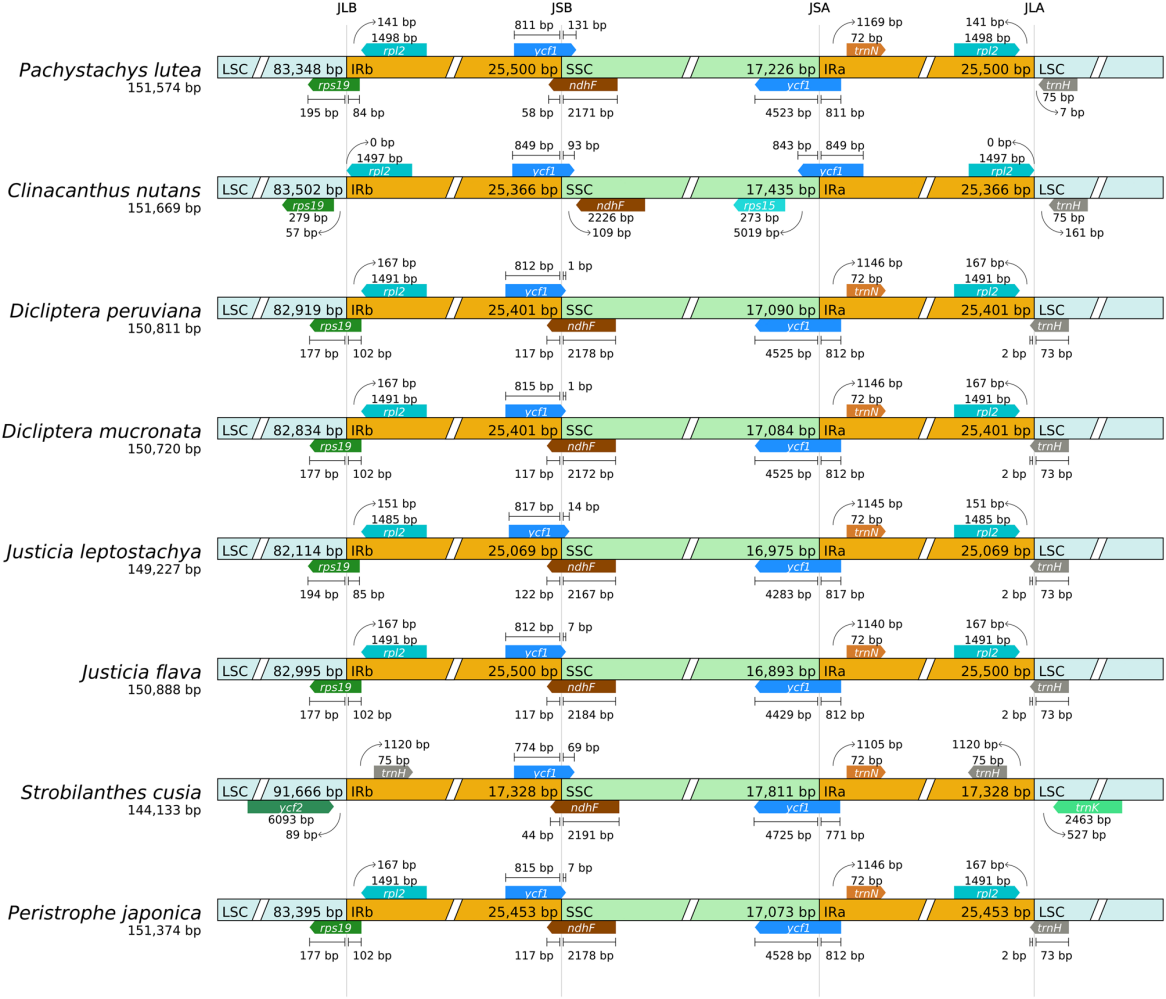
Comparison of the boundaries of LSC, IR and SSC among chloroplast genome of eight Acanthaceae species. The genes around the borders are shown above or below the main line. The JLB, JSB, JSA, and JLA represent junction sites of LSC/IRb, IRb/SSC, SSC/IRa, and IRa/LSC, respectively

The sequence of the IR boundary regions may extend outward and expand inward, resulting in changes in the copy number of related genes or the generation of pseudogenes in the boundary regions, which is a common phenomenon in the evolution of the cp. genome. In this study, we compared the boundary expansion and contraction of IR and SSC regions in eight species from six genus of the family Acanthaceae (Fig. 6). The results revealed little variation in the size of the cp. genomes across the eight species, with the exception of *S. cusia*, which exhibited a reduction of 5,094 to 7,536 bp compared to the other species. Similarly, the size of the IR regions of *S. cusia* showed a distinct shorter length. In the six species, *rps19* straddled the IRb/LSC (JLB) boundary (except *C. nutans* and *S. cusia*), *ndhF* straddled the IRb/SSC (JSB) (except *C. nutans*), and *ycf1* straddled the IRb/SSC (JSB) and the IRa/SSC (JSA). *rpl2* was located within the IR boundary (absent in *S. cusia*), *trnN* was located within the IR boundary (absent in *C. nutans*), and *trnH* was located near the LSC-IRa boundary junction. The changes in genes at the four boundaries of *P. lutea* were consistent with two species of *Dicliptera* genus, two species of *Justicia* genus and one species of *Peristrophe* genus. Compared with *C. nutans*, *rps19* at the JLB boundary expanded to the LSC region but contracted in the IRb direction, and *ndhF* showed obvious contraction in the IRb direction. The IR/SC borders were validated using Sanger sequencing [21], which was completely consistent with the sequencing results from the Illumina sequencing platform (Fig. S4).

### Analysis of cp. genome sequence variation

**Fig. 7:**
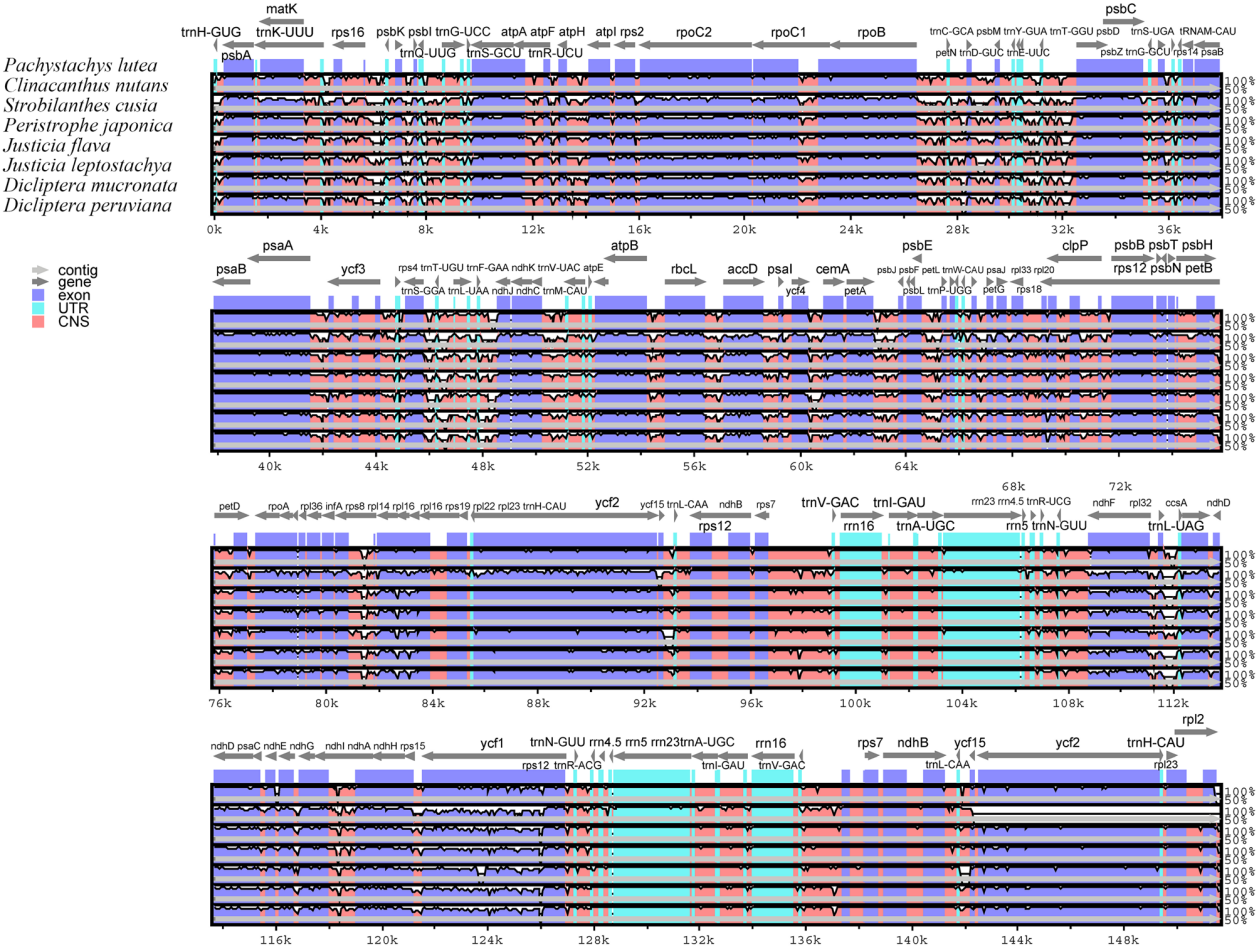
Global alignment analysis of chloroplast genome in eight Acanthaceae species. Annotated genes are displayed along the top. The horizontal axis indicates the coordinates in the chloroplast genome and the vertical axis indicates the percentage identity (between 50 and 100%). Genome regions are color-coded as exon, intron, and conserved non-coding sequences (CDS)

Visual analysis of the similarity among the eight cp. genome sequences showed that there was little difference in the gene interval composition of the cp. genomes of Acanthaceae plants, which was relatively consistent (Fig. 7). Among the four regions, the LSC region had the highest variability in changes, followed by SSC region, and the IR regions had the lowest variability and was the most conserved, which was consistent with the results of boundary analysis. From the perspective of the non-gene coding regions and gene coding regions, the degree of variation in the non-gene coding regions was relatively high, while the gene coding regions were relatively conserved. However, there was a significant difference in the degree of variation in the gene coding regions, such as those of *rps19*, *ycf1*, *ndhF*, *ndhA*, *clpP*, and *ycf3*. This indicated the existence of single nucleotide polymorphisms (SNPs) in the coding regions of genes such as *rps19*, *ndhF* and *ycf1*, which was further confirmed by gene sequence comparison (Fig. S5).

### Substitution rates of PCGs

**Fig. 8:**
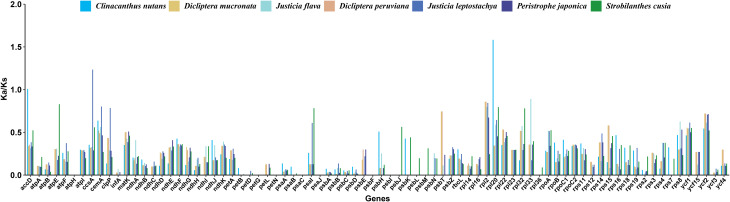
Ka/Ks values of selected PCGs of *P. lutea* with close species. The x-axis represents genes, and the order is alphabetical, while the y-axis represents the Ka/Ks value of each gene

The non-synonymous substitutions (Ka)/synonymous substitutions (Ks) ratio in genetics is used to evaluate the existence of selection pressure on a certain PCG during evolution. A Ka/Ks value greater than 1 implies positive selection. A Ka/Ks value of 1 suggests neutral selection. When the Ka/Ks ratio is below 1, it signifies negative selection [22]. Selected 80 PCGs from *P. lutea* cp. genome were compared with seven species from Acanthaceae for Ka/Ks calculation (Fig. 8; Table S3). Overall, most genes of *P. lutea* cp. genome experienced negative selection throughout evolution, as shown by the Ka/Ks values of 77 PCGs, being less than 1 when compared to other seven species. The high Ka/Ks values of *rpl20* from *P. lutea* compared to *C. nutans* suggested that positive selection occurred during evolution, meanwhile, *accD* also shows signs of positive selection. In addition, when *P. lutea* is compared with *J. leptostachya*, *ccsA* exhibits positive selection.

### Phylogenetic analysis

**Fig. 9:**
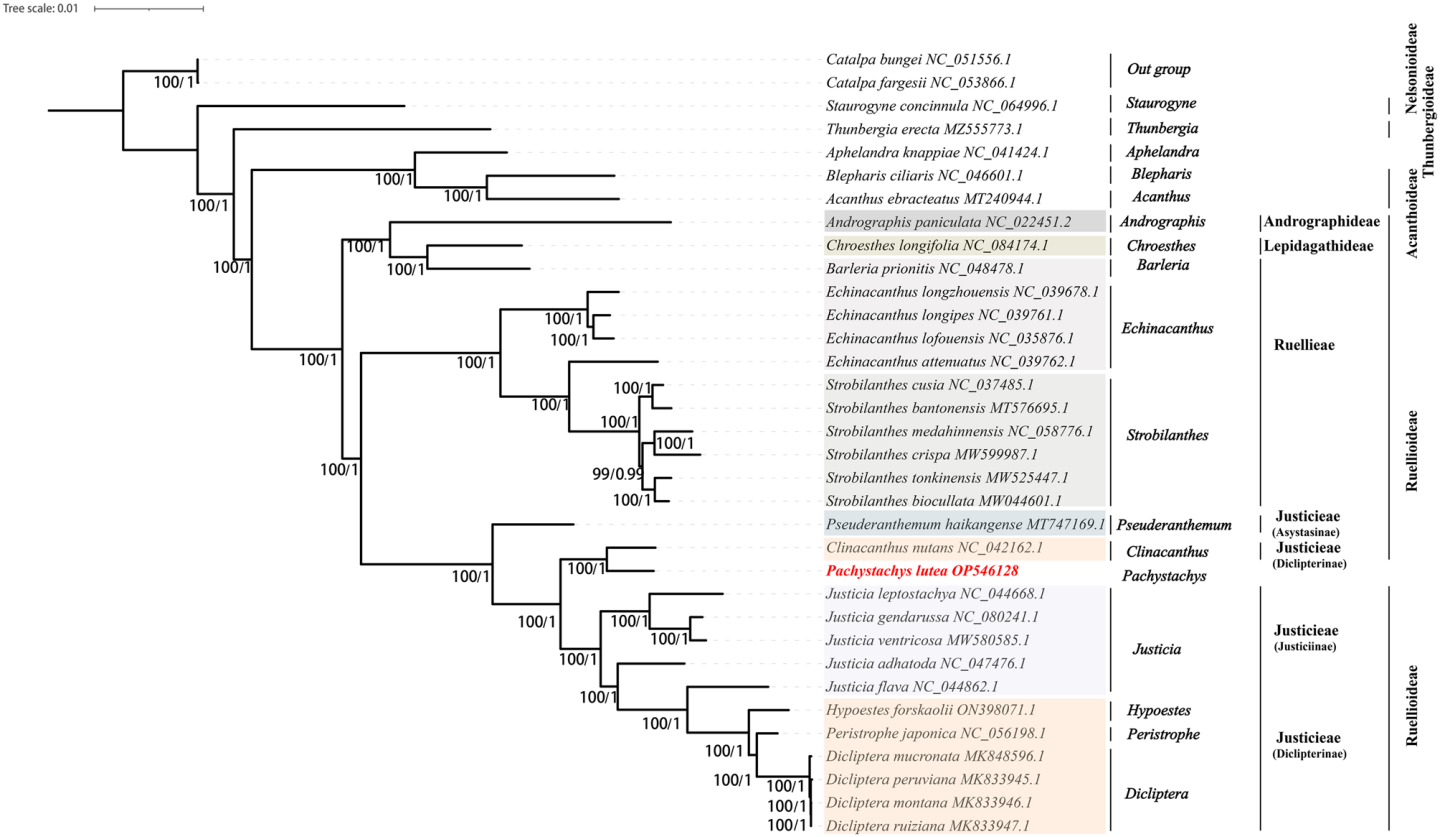
The phylogenetic tree of *P. lutea* was constructed by ML method based on the analysis of conserved PCGs of 34 species

To elucidate the evolutionary dynamics within the family Acanthaceae, particularly focusing on *P. lutea*, a comprehensive phylogenetic analysis was conducted, encompassing *P. lutea* and 34 other samples from 16 distinct genus, with two species assigned as outgroups for comparative context. This investigation was founded on the analysis of conserved PCGs (Fig. 9) and cp. genomes (Fig. S6), facilitating a dual perspective on phylogenetic relationships. Overall, both phylogenetic trees exhibited the same evolutionary pattern. Namely, the species from the *Pseuderanthemum*, *Clinacanthus*, *Pachystachys*, *Justicia*, *Hypoestes*, *Peristrophe*, and *Dicliptera* exhibited well-defined clustering, indicating a clear evolutionary relationship among them. *P. lutea* and *C. nutans* were found to be especially closely related, as evidenced by a bootstrap support (BS) value of 100, which strongly supports the robustness of this phylogenetic relationship.

## Discussion

The study of cp. genome sequences gives extensive information for phylogenetic research of plants, and DNA barcoding utilizing cp. markers allows for accurate identification of plant species [12]. In this study, the cp. genome of *P. lutea* was sequenced, assembled and annotated by high-throughput sequencing technology, and its genome structure, SSR sites, codon preference and phylogeny were analyzed. The findings will provide valuable insights into the evolutionary relationships and genetic variability of *P. lutea*, which is essential for its preservation. Genomic data may provide references for the development of plans for habitat restoration and the preservation of genetic reservoirs.

The complete cp. genome obtained had a total length of 151,574 bp, a total of 132 annotated genes, and a GC content of 38.18%. Compared with the reported homologous plants such as *C. nutans* (151,669 bp, 38.40%) [23], *D. peruviana* (150,811 bp, 38.00%) [24], *D. mucronata* (150,720 bp, 38.00%) [24], *J. flava* (150,888 bp, 38.20%) [25] and *P. japonica* (151,374 bp, 38.07%) [26], the Acanthaceae plants have highly similar genome sizes, structures, compositions, and GC contents, indicating that the plants of the Acanthaceae family exhibit good conservatism during evolution.

SSRs are widely distributed in most plants, mainly in the external and noncoding regions of genes, and are often used in species identification, genetic diversity analysis and molecular marker-assisted breeding [27]. In this study, a total of 47 SSR sites were found in the cp. genome of *P. lutea*, of which mononucleotides were the most abundant (85.11%), followed by dinucleotides (12.76%). The repetitive units of SSRs were mainly composed of A and T base combinations, with fewer G/C repeats. This further indicated that SSRs in cp. genomes exhibit significant AT preference, which may be related to the difficulty of AT and GC chain uncoupling. Combined with the results of cp. genome sequence variation analysis, the degree of variation in the IR regions with higher GC content was significantly lower than that in the LSC and SSC regions with lower GC content. Therefore, it can be inferred that base preference may be positively correlated with the degree of sequence variation, and it also indicated that the structure of the cp. genome of *P. lutea* was highly conserved. Consequently, the identified SSR loci hold great potential as they can offer a solid theoretical basis for the identification of *P. lutea*, phylogenetic analysis, and the development of molecular markers related to this particular plant species.

Codons serve as a crucial link among nucleic acids, proteins, and genetic material, thereby playing a significant role in the transmission of genetic information within organisms. Their preferred usage patterns offer dependable information for investigations into gene function, species evolution, and other related aspects [28, 29]. In the present study, Leu was identified as the most abundant amino acid, accounting for 10.71% within the cp. genome of *P. lutea*. The cp. genome of *P. lutea* demonstrated a preference for using codons ending with A/T. The occurrence of codon usage bias during the evolution of the cp. genome is attributed to natural selection and mutations [30]. It is well-documented that an ENc value greater than 40 implies a weak codon preference [31, 32]. Given that the GC3 contents were less than 50%, it could be inferred that the codons in this genome tended to utilize A and T bases. This finding was in alignment with the results of the codon preference analysis conducted on *J. flava*, suggesting that the closer the phylogenetic relationship between species, the more similar their codon usage preferences are. This further corroborated the conclusions put forward by Parvathy et al. [33]. Moreover, through Neutral-plot, ENC-plot, and PR2-plot analyses, it was determined that natural selection constitutes the primary factor influencing the codon usage bias observed in *P. lutea*.

The IR region of the cp. genome is considered to be the most conserved region and plays an important role in maintaining the stability of the cp. genome [34]. The contraction, expansion and deletion of IR boundaries can cause differences in cp. genomes [35]. Comparisons between LSC-IR and SSC-IR boundaries, and the genome sequence variation in the eight species’ complete cp. genomes showed that the LSC/SSC regions had higher variability, while the IR regions had lower variability, but the whole genome was still relatively conserved. In addition, there was variation among genomes, variation was manifested as the location and number of base pairs in the borders of four genes, *rps19*, *ndhF*, *ycf1*, and *trnH*. This may be caused by the instability of LSC-IR and SSC-IR boundaries and the different degrees of expansion and contraction during the historical evolution of species. The *rps19* gene is a key component of the ribosome biogenesis process, which is of vital importance for plant protein synthesis. Any variation in *rps19* may have an impact on the overall growth rate of the plant. The *ycf1* is located on the plastid membrane and promotes the transmembrane transport of various molecules. Mutations in *ycf1* will alter the plant’s response to environmental stress. The *ndhF* gene is associated with the NADH dehydrogenase-like complex, which is involved in the electron transport during photosynthesis. Changes in *ndhF* may affect the photosynthetic efficiency of the plant. These genetic variations may have enabled *P. lutea* to adapt to its native tropical habitats in South America. They may also have contributed to the successful cultivation of *P. lutea* in the southern regions of the Yangtze River Basin in China. Additionally, these regions with partial differences can provide molecular bases for the identification and phylogenetic analysis of different species in the family Acanthaceae.

Adaptive evolution has a profound implication on the study of structural and functional variation of genes, and Ka/Ks is an effective method to evaluate whether the adaptive evolution of PCGs has occurred [36]. Ks occurs more frequently than Ka in most genes of organisms, so Ka/Ks values are usually less than 1 [37]. In this study, we detected that the majority of genes in Acanthaceae species had Ka/Ks < 1, indicating that the cp. genes of Acanthaceae species had been subjected to strong purifying selection during the long evolutionary process. The Ka/Ks of photosynthetic related genes such as *atpE*, *ndhE*, *psbH* and *psbJ* were less than 1, indicating that they were subjected to strong purifying selection during the evolutionary process. While *rpl20*, *accD*, and *ccsA* showed positive selection in other species, indicating that the aforementioned genes had a strong influence on the evolution trend of different species. Furthermore, PCGs of the cp. genome encodes many critical proteins involved in photosynthesis and other metabolic processes, playing a role in the development of plant defense against pathogen ingress, stress tolerance, and ornamental traits [38, 39]. For example, the photosynthesis gene *psbA* encodes a critical and highly conserved component of the photosystem II reaction center, polypeptide D1, which is participated in the photosynthetic electron transport chain. Under conditions of high-light stress, the increased production of the *psbA* protein helps to protect the photosynthetic machinery from damage caused by excess light energy, thereby maintaining photosynthetic efficiency and ensuring the plant’s survival and growth. Thus, the transcription and translation of *psbA* play an important role in high-light stress responses [40]. Ribosomal proteins are essential for cell survival, among which *rps15* and *rpl33* are important components of the ribosome, responsible for protein synthesis in the cell, and their absence is also causally responsible for the high chilling sensitivity in plants [41]. For example, tobacco plants lacking the ribosomal proteins *rps15* or *rpl33* exhibited heightened sensitivity to cold stress [42]. In this study, *psbA*, *rps15* and *rpl33* genes were found in cp. genome of *P. lutea*, which may play a role in photosynthesis and resistance. The *psbA* gene can maintain highly efficient photosynthesis under the intense light conditions typical of tropical regions, ensuring a stable energy supply for the plants. Adequate energy serves as the cornerstone for the growth of *P. lutea* and also contributes to its continuous blooming. With stable energy input, *P. lutea* can sustain the metabolic activities necessary for flower production throughout the year. Tropical regions are characterized by frequent fluctuations in temperature, humidity, and light intensity. The *rps15* and *rpl33* genes facilitate rapid environmental adaptation of *P. lutea* by promoting efficient protein synthesis. When these two genes function properly, *P. lutea* can synthesize proteins in a timely manner to cope with environmental changes. This significantly enhances the plant’s tolerance to various environmental stresses, thereby providing support for its year-round growth and blooming. Overall, the genetic variations in the cp. genome of *P. lutea*, especially the presence and functionality of the *psbA*, *rps15*, and *rpl33* genes, may represent crucial factors underlying the unique growth and blooming characteristics of *P. lutea*.

The family Acanthaceae has many species and genera, wide distribution, outstanding morphological diversity, including shrubs, herbs, and even vines, and outstanding habitat diversity. For a long time, the family was considered difficult to study. Since 1789, when French botanist Antoine-Laurent de Jussieu (1789) [43] proposed the natural classification of plants according to the relative positions of stamens and ovaries, the family Acanthaceae was published. Many scholars, such as Nees (1832) [44], Bentham (1876) [45], Lindau (1895) [46], and Bremekamp (1965) [47] have proposed different classification systems according to different classification characteristics. In recent years, the study of Acanthaceae evolutionary biology has benefited greatly from the utilization of cp. genomes. Gao et al. (2019) [1] determined the cp. genomes of four *Echinacanthus* species and resolved the phylogenetic relationship within Acanthaceae, which exhibited that *Echinacanthus* was sister to *Strobilanthes*. Similarly, Huang et al. (2020) [24] demonstrated that *Justicia*, *Clinacanthus*, and *Dicliptera* belong to one branch, whereas *Echinacanthus* and *Strobilanthes* belong to another branch. Our findings were consistent with the evolutionary connection among the five aforementioned taxa. Furthermore, our study identified the position of *P. lutea* in Acanthaceae for the first time. That is, *P. lutea* was closely related to members of Justiciinae, but it was most closely related to *C. nutans* of Diclipterinae, and they clustered into a small branch to form a sister relationship with a support value of 100%. McDade et al. (2000) [48] classified *Pachystachys* and *Clinacanthus* into the same subtribe. In this study, it was found that there was a close relationship between *P. lutea*, Justiciinae and Diclipterinae. As a monospecific genus, *Pachystachys* was suggested to be included in the Justicieae, but whether it can be divided into Justiciinae or Diclipterinae can be further discussed in future studies.

## Conclusions

As a newly emerged species resulting from natural selection during the evolutionary process of the Acanthaceae family, *P. lutea* boasts a genome structure that is highly conserved and bears a remarkable similarity to those of other related species. Phylogenetic analysis has revealed that *P. lutea* shares a close kinship with *C. nutans*. Moreover, it can be grouped together with species from *Justicia*, *Hypoestes*, *Peristrophe*, and *Dicliptera* within the Justicieae tribe. The cp. genome data obtained in this research are of significant importance for the conservation as well as the rational development of the germplasm resources of *Pachystachys*, thus playing a crucial role in safeguarding the biodiversity and facilitating the sustainable utilization of these valuable genetic materials within the Acanthaceae family. Subsequent research will focus on validating the functions of the adaptive genes identified in the cp. genome of *P. lutea*. These genomic data can directly serve conservation and breeding efforts. By leveraging the identified genetic variations, it is promising to cultivate new varieties with outstanding ornamental traits, strong stress resistance, and excellent ecological adaptability, thus promoting the diverse applications of plants in the Acanthaceae family.

## Materials and methods

### Genomic DNA extraction and sequencing

Fresh leaves of *P. lutea* were collected from Xinyang, Henan Province, China (the experimental base of Xinyang Agriculture and Forestry University: 114° 12’ E, 32° 16’ N, altitude: 102 m). The leaves were frozen in liquid nitrogen before DNA extraction. Total genomic DNA was extracted from leaves using the CTAB method [49] and was sent to Shanghai Origingene Biotechnology Co., Ltd. for DNA library construction. The DNA library was constructed according to the instructions of the TruSeq DNA Sample Prep Kit (Illumina, USA), with the average insert length of 300–380 bp. Then, the samples were sequenced by using the Illumina NovaSeq 6000 sequencing platform (Illumina, San Diego, CA) with paired-end reads of 150 base pairs (insert size of 180–250 bp). A total of 8.9 GB raw data were produced and deposited in the SRA database (Sequence Read Archive, http://www.ncbi.nlm.nih.gov/Traces/sra).

### Genome assembly and annotation

The quality of the raw paired-end reads was assessed using FastQC v0.11.7 [50] software. To ensure the accuracy of subsequent biological information analysis, the sequencing data for adapter sequences, low-quality reads, sequences with a high N rate, and sequences with insufficient lengths included in the raw data reads were filtered to obtain high-quality clean reads. After the quality evaluation, the reads were assembled by using both Fast-plast v.1.2.8 (https://github.com/mrmckain/Fast-Plast) [51]. Single contigs containing the complete cp. genome were generated. PGA and Geseq were used for gene prediction and annotation of the *P. lutea* cp. genome [52, 53], with default parameters and percent identity cut-off for PCGs and RNAs set at ≥ 60 and ≤ 85, respectively. All sample annotation results were manually corrected. BWA (v.0.7.17-r1188) was used to process comparison data, SAMtools (v.1.9) was used to calculate coverage depth, and then visualized using ggplot2 in R [54]. After the annotation was completed, the sequence of the cp. genome of *P. lutea* was deposited in the GenBank database with accession number OP546128. CPGview (http://www.1kmpg.cn/cpgview/) was used to generate a circular cp. genome map [55].

### Simple sequence repeats analysis

MISA v1.0 software [56] was used for SSRs analysis in the cp. genome of *P. lutea*, and the number of repetitions was set to 10, 6, 5, 5, 5, and 5 from mononucleotide to hexanucleotide, respectively. The maximum cardinality between any two SSRs was set to 100 bp. MISA was also used to determine the specific location of SSRs in the cp. genome of *P. lutea*.

### Codon preference analysis

The PCGs sequence of cp. genome in *P. lutea* were manually screened to remove duplicates and coding sequences less than 300 bp in length, and the eligible sequences were used for subsequent analysis. Using CodonW V1.4.4 software [57] and the online software CUSP (http://emboss.toulouse.inra.fr/cgi-bin/emboss/cusp) [58], the ENC, GC content of codon 1, 2 and 3 bases (GC1, GC2, GC3, respectively) and RSCU were calculated. Additionally, Neutral-plot, ENC-plot, and PR2-plot analysis were performed.

### Sequence variation map and variations of IR regions sequences

The comparison between the genome of *P. lutea* and seven Acanthaceae species’ cp. genomic sequences was performed using the mVISTA program (http://genome.lbl.gov/vista/mvista/submit.shtml) to find interspecific variation [59]; the annotation of *P. lutea* was used as a reference in the Shuffle-LAGAN mode. Furthermore, comparisons between the borders of the IR (IRa and IRb), the SSC and LSC regions were generated using the CPJSdraw software [60]. The IR/SC borders of the *P. lutea* were validated using Sanger sequencing [61].

### Ka/Ks analysis

The Ka/Ks substitution rates of the PCGs in *P. lutea* cp. genome were compared with seven related species from Acanthaceae. Homologous gene pairs from eight species were extracted and aligned using MAFFT v7.427 (https://mafft.cbrc.jp/alignment/software/) [62]. After alignment, the Ka and Ks values for each gene pair were calculated with KaKs_Calculator v2.0 (https://sourceforge.net/projects/kakscalculator2/) using the MLWL method [63]. Finally, the Ka/Ks ratios of each gene pair were summarized and plotted using the R package ggplot2.

### Phylogenetic analysis

In this study, to determine the phylogenetic position of *P. lutea*, the complete cp. genomes of 33 species were downloaded from NCBI. Among these, 31 from four subfamilies within the Acanthaceae family, and two (*Catalpa bungei* and *Catalpa fargesii*) from outside this family, serving as outgroup species. The Multiple sequence alignment of the 34 species including *P. lutea* was performed by using MAFFT [62] software and Gblock software [64] to find the conserved sequences between different species. The best substitution model, GTR + G, was selected in the jModelTest v2.1.7 program [65]. The phylogenetic tree was constructed with the maximum-likelihood (ML) method using RAxML v8.2.12 software [66]. Bootstrap analysis was used to evaluate the support for individual clades with 1000 replicates.

## Supplementary Information

Below is the link to the electronic supplementary material.

**Supplementary Material 1: Fig. S1.** Overall coverage depth of the chloroplast genome assembly of *P. lutea*

**Supplementary Material 2: Fig. S2.** (**A**) Schematic map of the cis-splicing genes in the *P. lutea* chloroplast genome. The genes are arranged from top to bottom based on their order on the chloroplast genome. The gene names are shown on the left, and the gene structures are on the right. The exons are shown in black; the introns are shown in white. The numbers below the arrows denote the positions of the gene boundaries within the genome. The arrow indicates the sense direction of the gene. (**B**) Schematic map of the trans-splicing gene *rps12* in the *P. lutea* chloroplast genome. It illustrates the transcription process of *rps12*. Transcript 1 and Transcript 2 depict two different transcription patterns. Genome (+) and (−) indicate the positive and negative strands of the genome, respectively. It has three unique exons. Two of them are duplicated as they are located in the IR regions

**Supplementary Material 3: Fig. S3.** Dotplot of self-comparison of the chloroplast genome of *P. lutea* following Fast-plast assembly

**Supplementary Material 4: Fig. S4.** The chromatogram of IR/SC boundary sequence generating using Sanger sequencing

**Supplementary Material 5: Fig. S5.** Alignments of the amino acid sequences of *rps19* (**A**), *ndhF* (**B**) and *ycf1* (**C**) among eight Acanthaceae species

**Supplementary Material 6: Fig. S6.** The phylogenetic tree of *P. lutea* was constructed by ML method based on the complete chloroplast genome sequences of 34 species

**Supplementary Material 7: Table S1.** The SSR loci distribution of the chloroplast genome in *P. lutea*

**Supplementary Material 8: Table S2.** RSCU analysis of each amino acid in *P. lutea*

**Supplementary Material 9: Table S3.** Ka/Ks values of PCGs of *P. lutea* with close species

## Data Availability

The original sequencing data have been submitted to the NCBI database and received GenBank accession number OP546128. The data used in this study are available in the public domain (https://www.ncbi.nlm.nih.gov). And the associated Bio-project, SRA, Bio-sample numbers are PRJNA884896, SRX17730640, and SAMN31059961, respectively.

## Declarations

### Statement

Our experimental research and field studies on plants comply with relevant institutional, national, and international guidelines and legislation.

## Abbreviations

cp.: Chloroplast
LSC: Large single-copy
SSC: Small single-copy
IR: Inverted repeat
PCGs: Protein-coding genes
SSRs: Simple sequence repeats
ML: Maximum-likelihood
ITS: Internal transcribed spacers
ENC: Effective number of codons
GC1, GC2, GC3: GC content of codon 1, 2 and 3 bases
RSCU: Relative synonymous codon usage
Ka: Non-synonymous substitutions
Ks: Synonymous substitutions
Leu: Leucine
Cys: Cysteine
Met: Methionine
Trp: Tryptophan
JLB: IRb/LSC
JSB: IRb/SSC
JSA: IRa/SSC
JLA: IRa/LSC
SNPs: Single nucleotide polymorphisms
BS: Bootstrap support
