# Supplementary figures and images for: Complete chloroplast genome sequence of Pachystachys lutea Nees: genome structure, adaptive evolution, and phylogenetic relationships
Source: BMC Genom Data. 2025 Nov 19;26:87. doi: 10.1186/s12863-025-01380-9 (PMC12628888; doi:10.1186/s12863-025-01380-9)

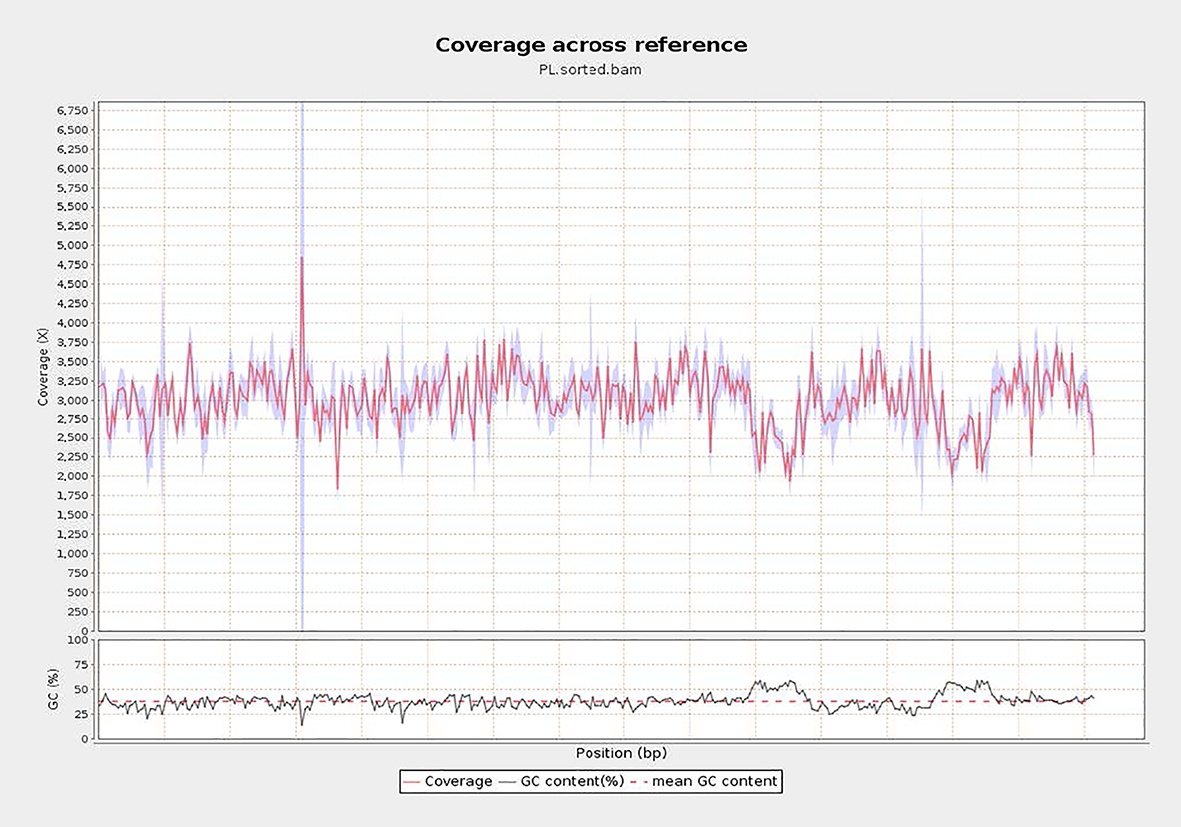

Supplement: Supplementary file 1 — Supplementary Material 1: Fig. S1. Overall coverage depth of the chloroplast genome assembly of P. lutea [file 12863_2025_1380_MOESM1_ESM.tif]

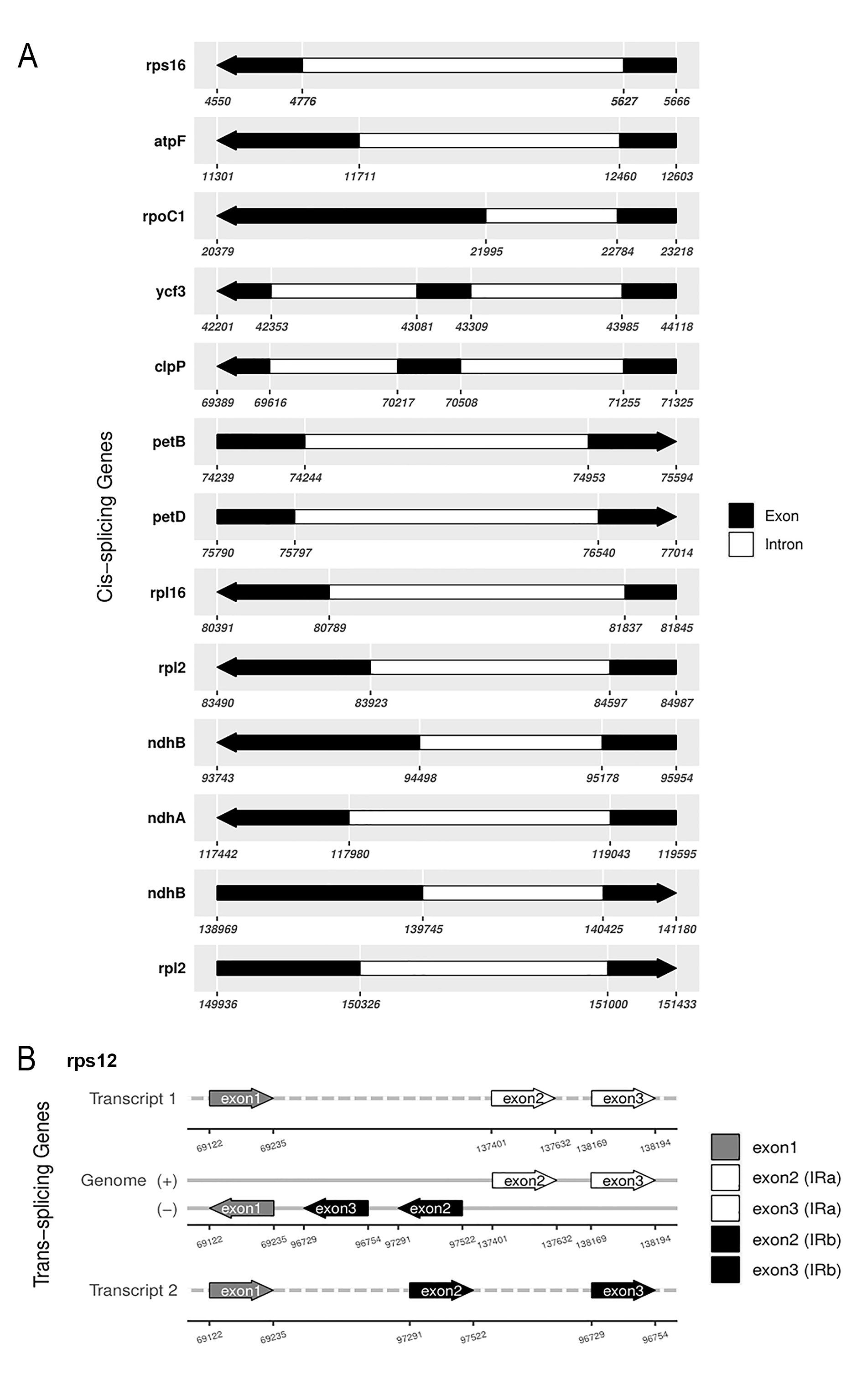

Supplement: Supplementary file 2 — Supplementary Material 2: Fig. S2. (A) Schematic map of the cis-splicing genes in the P. lutea chloroplast genome. The genes are arranged from top to bottom based on their order on the chloroplast genome. The gene names are shown on the left, and the gene structures are on the right. The exons are shown in black; the introns are shown in white. The numbers below the arrows denote the positions of the gene boundaries within the genome. The arrow indicates the sense direction of the gene. (B) Schematic map of the trans-splicing gene rps12 in the P. lutea chloroplast genome. It illustrates the transcription process of rps12. Transcript 1 and Transcript 2 depict two different transcription patterns. Genome (+) and (−) indicate the positive and negative strands of the genome, respectively. It has three unique exons. Two of them are duplicated as they are located in the IR regions [file 12863_2025_1380_MOESM2_ESM.tif]

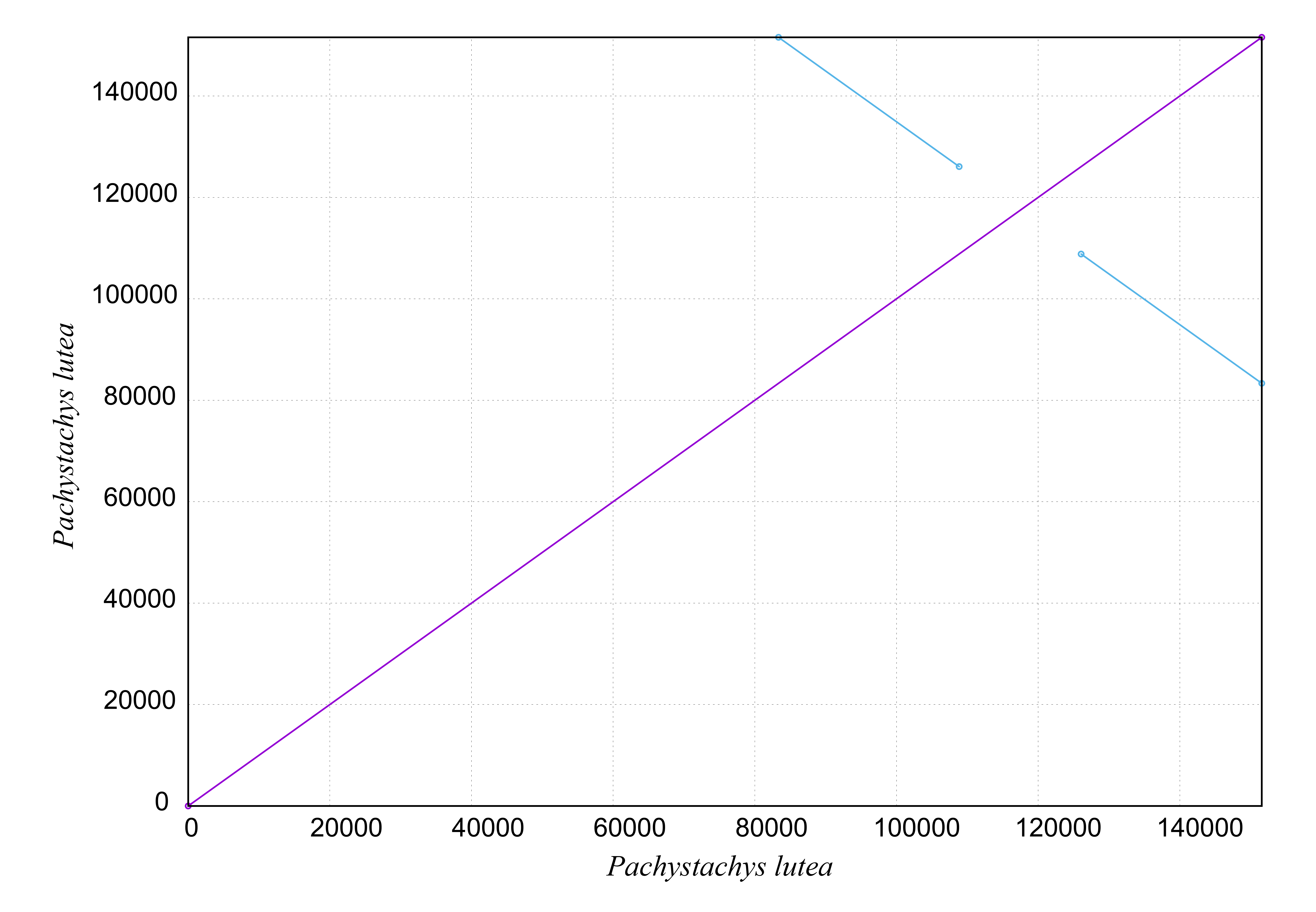

Supplement: Supplementary file 3 — Supplementary Material 3: Fig. S3. Dotplot of self-comparison of the chloroplast genome of P. lutea following Fast-plast assembly [file 12863_2025_1380_MOESM3_ESM.tif]

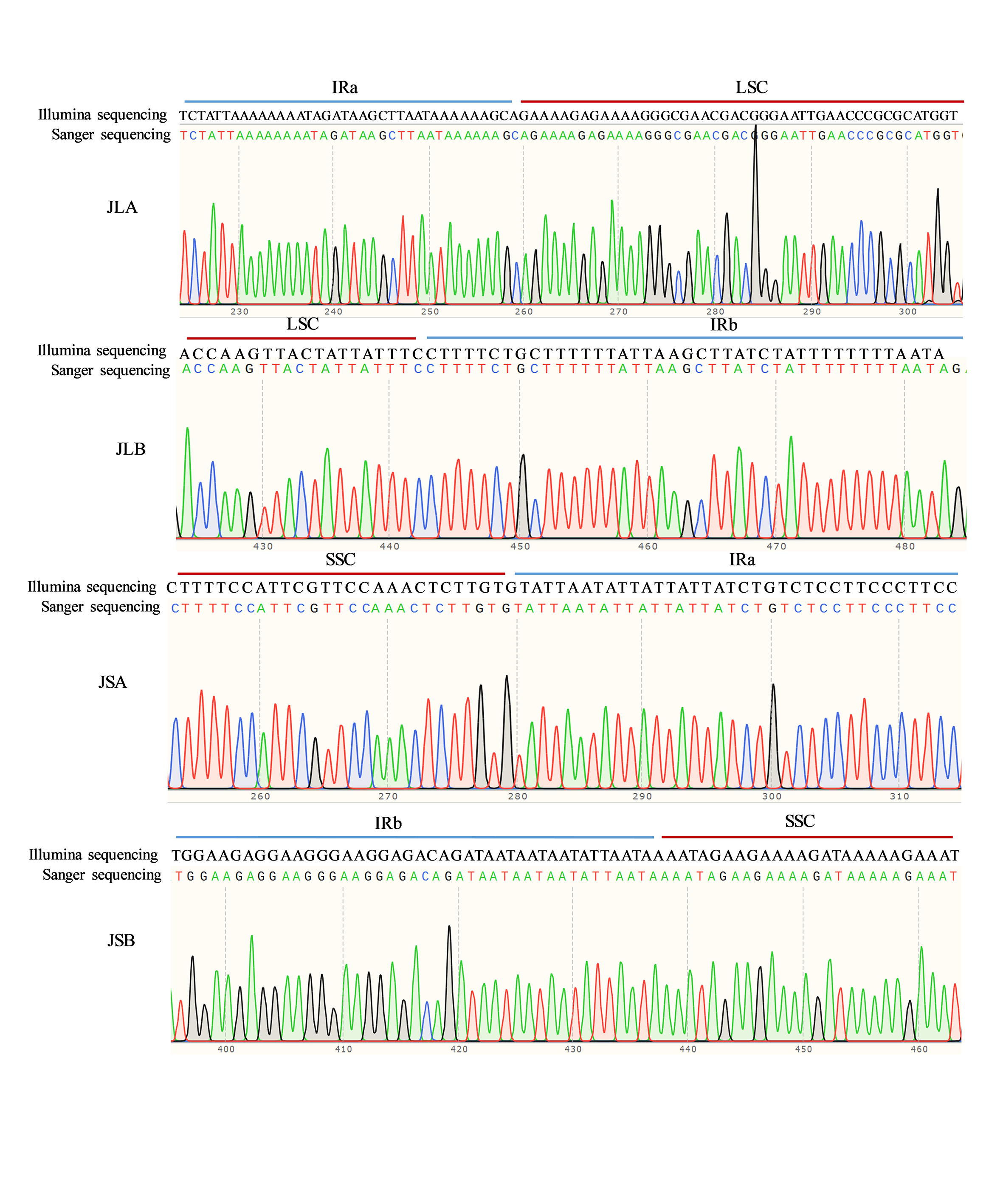

Supplement: Supplementary file 4 — Supplementary Material 4: Fig. S4. The chromatogram of IR/SC boundary sequence generating using Sanger sequencing [file 12863_2025_1380_MOESM4_ESM.tif]

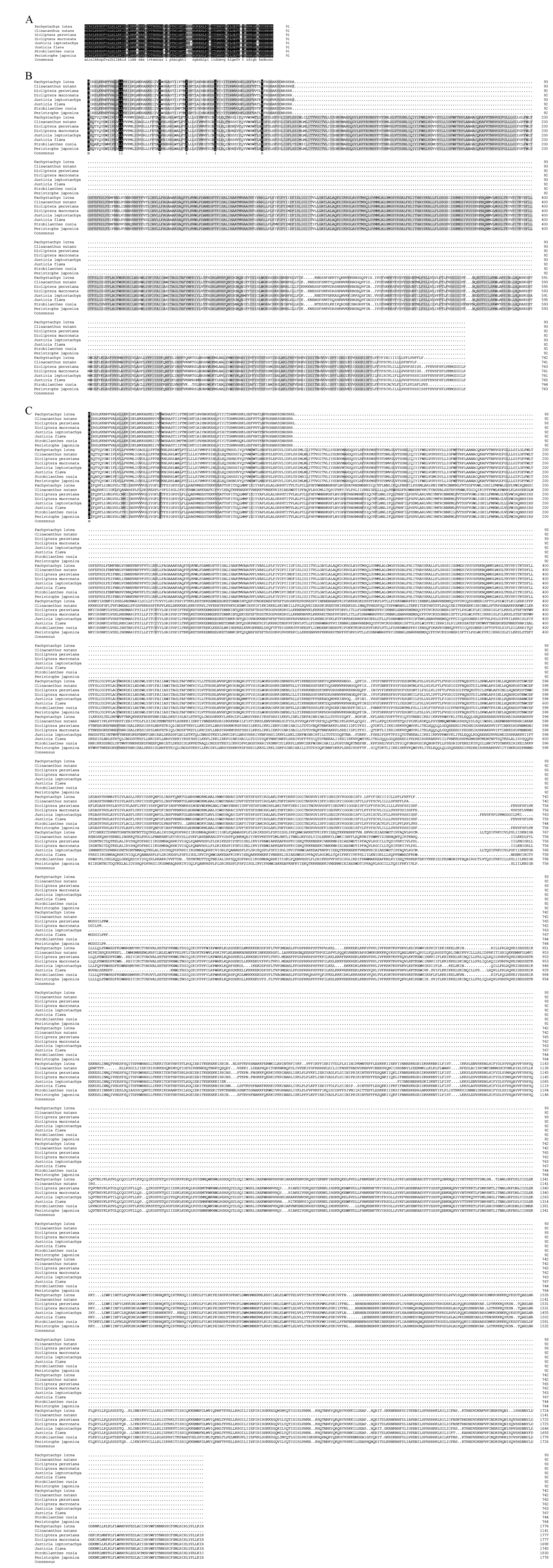

Supplement: Supplementary file 5 — Supplementary Material 5: Fig. S5. Alignments of the amino acid sequences of rps19 (A), ndhF (B) and ycf1 (C) among eight Acanthaceae species [file 12863_2025_1380_MOESM5_ESM.tif]
